# Comprehensive assessment of left atrial and ventricular remodeling in paroxysmal atrial fibrillation by the cardiovascular magnetic resonance myocardial extracellular volume fraction and feature tracking strain

**DOI:** 10.1038/s41598-021-90117-6

**Published:** 2021-05-25

**Authors:** Akimasa Yamada, Naoki Hashimoto, Hidesato Fujito, Takumi Hatta, Yuki Saito, Naoto Otsuka, Yuji Wakamatsu, Masaru Arai, Ryuta Watanabe, Sayaka Kurokawa, Daisuke Kitano, Koichi Nagashima, Shunichi Yoda, Yasuo Okumura

**Affiliations:** Department of Cardiovascular Medicine, Nihon University Graduate School, 30-1 Oyaguchi kamimachi, Itabashi, Tokyo 173-8610 Japan

**Keywords:** Cardiology, Medical research

## Abstract

Atrial fibrillation (AF) is a progressive disease that starts with structural or functional changes in the left atrium and left ventricle, and evolves from paroxysmal toward sustained forms. Early detection of structural or functional changes in the left atrium and left ventricle in the paroxysmal stage could be useful for identifying a higher risk of progression to persistent AF and future cardio-cerebrovascular events. The aim of this study was to test the hypothesis that the feature tracking (FT) left atrial (LA) strain and left ventricular (LV) extracellular volume fraction (ECV) derived from cardiovascular magnetic resonance (CMR) could detect early changes in remodeling of the left atrium and ventricle in the paroxysmal AF (PAF) stage. The participants were comprised of 106 PAF patients (age, 66.1 ± 10.7 years; 66% male) who underwent clinical CMR before pulmonary vein isolation and 20 control subjects (age, 68.3 ± 8.6 years; 55% male). The CMR-FT LA strain/phasic function and LV-ECV were compared between the PAF and control groups. The total and passive LA empty fraction (LAEF) and LA strain (corresponding to LA reservoir and conduit function) were decreased in the PAF group as compared to the control group. However, active LAEF (corresponding to the LA booster pump function) did not differ significantly between the PAF group (33.9 ± 10.9%) and control group (37.9 ± 13.3%, p = 0.15), while the active LA strain (corresponding to the LA booster pump function) was significantly decreased in the PAF group (11.4 ± 4.3 vs. 15.2 ± 5.6%, p = 0.002). The LV-ECV was significantly greater in the PAF group (28.7 ± 2.8%) than control group (26.6 ± 2.0%, p = 0.002). In the PAF group, the LV-ECV correlated significantly with the E/e′ and LA volume index. Regarding the LA strain, correlations were seen between the LV-ECV and both the reservoir function and conduit function. CMR-FT LA strain in combination with the LV-ECV in a single clinical study offers a potential imaging marker that identifies LA/LV remodeling including subtle LA booster pump dysfunction undetectable by the conventional booster pump LAEF in the PAF stage.

## Introduction

Atrial fibrillation (AF) is the most common clinical arrhythmia, with an increasing prevalence worldwide, and contributes to morbidity and mortality^1,2^. Our understanding of the left atrial (LA) structure and function has advanced significantly over the past decade with the marked development of multimodality imaging^3^. Accurate evaluation of the LA structural and functional remodeling is potentially key to the optimal management of AF in clinical practice.

Cardiovascular magnetic resonance (CMR) is accurate, reproducible, and widely regarded as the noninvasive reference standard for structural, functional, and tissue-characterizing assessments of the atrium and ventricle^4,5^. CMR can determine the extracellular volume fraction (ECV) of the left ventricular (LV) myocardium, a hallmark of myocardial fibrosis, by measuring the T1 relaxation times before and after administration of gadolinium contrast^6^. An excellent correlation between the myocardial ECV derived from CMR and quantitative histopathology has been confirmed in a number of studies^7,8^. Feature tracking (FT) represents a novel method to quantify the myocardial strain directly from standard cine images without the need for complex and time-consuming sequences^9^. With the advent of the FT technology, myocardial strain can now be assessed from routine CMR studies, overcoming several inherent limitations of speckle-tracking echocardiography^10^. This novel approach has also recently been applied to assess atrial strain with its high spatial resolution and excellent endocardial border detection^11–14^. Using these advanced approaches, CMR could have the potential to quantify the LA strain and LV-ECV in a single study.

AF usually presents in a self-terminating paroxysmal form. The type of AF often progresses to become more sustained over time and LA remodeling concomitantly develops^15^. LA strain is an important imaging marker that correlates with functional LA remodeling and the risk of cardio-cerebrovascular events^16^. A large body of literature has been published on the relationship between the progression of LA remodeling and persistent AF^17–19^. Despite an intensive investigation into persistent AF, few studies have explored the characteristics of LA remodeling and LV myocardial fibrosis, particularly in the stage of paroxysmal AF (PAF). The present study therefore aimed to clarify whether the feature tracking LA strain and LV-ECV derived from a single clinical CMR study could detect the early changes in the remodeling of the left atrium and ventricle in the PAF stage.

## Methods

### Study population

We conducted a prospective, single-center, observational study. Consecutive patients with PAF who were scheduled for pulmonary vein isolation (PVI) at our institution between July 2018 and September 2020 were recruited for this study. PAF was defined as AF that spontaneously terminates within 7 days. Inclusion criteria for the PAF group were: (1) patients scheduled for first PVI; (2) patients in sinus rhythm at the time of attendance at the outpatient clinic. Exclusion criteria were as follows: (1) severe renal failure (glomerular filtration rate < 30 mL/m^2^); (2) severe valvular heart disease (e.g., severe aortic stenosis and regurgitation, and severe mitral regurgitation); (3) cardiomyopathy (e.g., hypertrophic cardiomyopathy, dilated cardiomyopathy, cardiac amyloidosis, cardiac sarcoidosis or iron overload); (4) myocardial infarction (by either history according to the presence of pathological Q waves on electrocardiography or wall motion abnormality on echocardiography). Patients were referred for CMR to image the pulmonary vein and screen for cardiac diseases. Twenty age- and sex-matched subjects with no history of cardiovascular disease and with normal results from physical examination, electrocardiography and echocardiography were recruited as healthy control subjects. This study was conducted in accordance with the principles of the Declaration of Helsinki and with the approval of the Institutional Review Board (Nihon University Itabashi Hospital Clinical Research Judging Committee: approval number RK-180410-02). Written informed consent for study participation was obtained from each patient.

### CMR protocol

MR images were acquired using a 1.5-T scanner (Ingenia; Philips Healthcare, Eindhoven, the Netherlands) with retrospective electrocardiographic gating. Sequences were acquired during breath-holds in the supine position. The comprehensive CMR protocol consisted of standard steady-state free precession (SSFP) cine MRI, T1 mapping by a modified look-Locker inversion recovery sequence (MOLLI) before contrast, late gadolinium enhancement (LGE) MRI, and T1 mapping by MOLLI after contrast. Standard SSFP cine images included coverage of the entire LV and LA using short-axis slices and 2-, 3-, and 4-chamber views with temporal resolution < 40 ms. LGE imaging was acquired with a T1-weighted inversion recovery gradient-echo sequence 15 min after contrast administration (0.15 mmol/kg, Gd-BTDO3A, Gadovist; Bayer Japan, Tokyo, Japan) in three long-axis slices (two-, three-, and four-chamber) and a stack of short-axis slices completely encompassing the LV. To calculate ECV, T1 measurements were acquired in a single breath-hold MOLLI sequence in three short-axis (basal, mid-ventricular, and apex) and two long-axis slices before and 20 min after contrast administration. Detailed CMR sequence parameters are available as supplemental materials.

### Image analysis

All images were analyzed by a blinded observer using commercial post-processing software (Circle CVI 42; Circle Cardiovascular Imaging Inc., Calgary, Canada).

### Volumetric and functional analyses

Endo- and epicardial LV contours were manually drawn in short-axis cine images covering from the mitral valve to the apex at end-diastole and end-systole to calculate end-diastolic and end-systolic volume, stroke volume, and ejection fraction. LV mass was calculated as the sum of myocardial volumes multiplied by the specific gravity (1.05 g/mL) of myocardial tissue. Papillary muscles were excluded from LV mass. LA volumes (LAV) were calculated using the biplane area-length method as previously described^20^. LAV were assessed at end-systole, just before opening of the mitral valve (LAV_max_), at end-diastole just before closure of the mitral valve (LAV_min_) and at diastole just before LA contraction (LAV_pre-ac_). In addition, LA total, passive, and active emptying fraction (EF) were calculated with the following formulas:^21^.

$$ \begin{aligned} & {\text{total}}\;{\text{LAEF }} =  \left( {{\text{LAV}}_{{{\text{max}}}} -{\text{ LAV}}_{{{\text{min}}}} } \right){\text{/LAV}}_{{{\text{max}}}} \times \, 100, \\ & {\text{passive}}\;{\text{LAEF }} =  \left( {{\text{LAV}}_{{{\text{max}}}} -{\text{ LAV}}_{{\text{pre-ac}}} } \right){\text{/LAV}}_{{{\text{max}}}} \times \, 100, \\ & {\text{active}}\;{\text{LAEF }} =  \left( {{\text{LAV}}_{{\text{pre-ac}}} -{\text{ LAV}}_{{{\text{min}}}} } \right){\text{/LAV}}_{{\text{pre-ac}}} \times \, 100. \\ \end{aligned} $$

## Feature tracking strain analysis

### Left ventricular strain

The images were analyzed with CMR analysis software that allowed for the measurement of 2 dimensional strain parameters based on standard cine SSFP images. Endo- and epicardial borders were semi-automatically drawn at end-diastole in short- and long-axis cines, excluding papillary muscles from the endocardial contour, then automatically propagated to all slices throughout the cardiac cycle. Tracking was visually reviewed and manually corrected in case of inaccurate automated border tracking. Short-axis cines were tracked to derive radial and circumferential strain, while 2-, 3-, and 4-chamber-view cines were tracked to derive longitudinal strain.

### Left atrial strain

**Figure 1:**
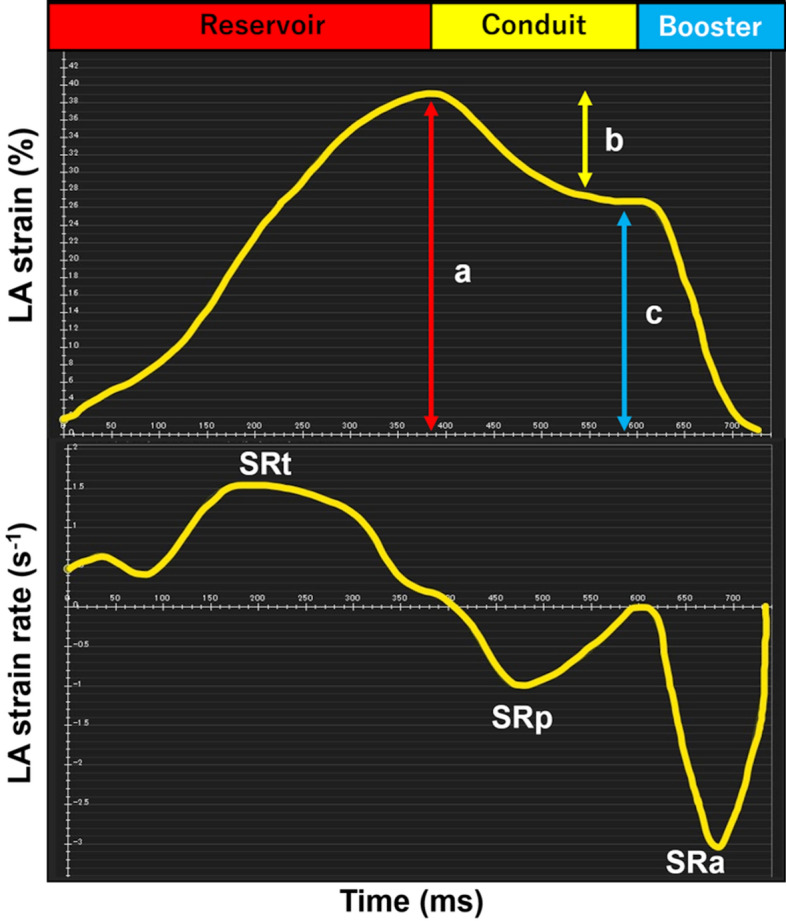
Quantification of left atrial strain and strain rate. Left atrial function comprised three components: reservoir function; conduit function; and booster pump function. Total strain (a) and peak positive strain rate (SRt) correspond to reservoir function. Passive strain (b) and peak early negative strain rate (SRp) correspond to conduit function. Active strain (c) and peak late negative strain rate (SRa) correspond to booster pump function.

At end diastole, endo- and epicardial borders were manually traced in 4- and 2-chamber views before the automated tracking algorithm was applied (pulmonary veins and LA appendage were excluded). Tracing was blindly repeated three times in 4- and 2-chamber views, and the results of LA strain and strain rate (SR) from the three repetitions were averaged in both views. As previously described^11^, three aspects of atrial strain were derived from strain curve: passive strain (corresponding to atrial conduit function), active strain (corresponding to atrial booster pump function) and total strain (corresponding to atrial reservoir function). Accordingly, three SR parameters were also calculated: peak early negative strain rate (SRp, corresponding to atrial conduit function), peak late negative strain rate (SRa, corresponding to atrial booster pump function) and peak positive strain rate (SRt, corresponding to atrial reservoir function) (Fig. 1).

### LV-ECV calculation

To calculate LV-ECV, native T1 and post-contrast T1 values were measured by drawing LV endocardial and epicardial borders with care to avoid the blood pool contamination on a series of three short-axis views before and after contraction administration. LV-ECV was then calculated as follows:^22^ ECV = (1 – hematocrit) × {[(1/T1_myocardium.post_) – (1/T1_myocardium.pre_)]/[(1/T1_blood.post_) – (1/T1_blood.pre_)]} × 100. The blood sample for hematocrit measurements was taken on the day of the CMR study (pre-CMR examination).

### Postablation follow-up

Follow-up was performed at our outpatient clinic and 12-lead electrocardiograms were recorded at 2 weeks, 1 month, and every 3 months after the catheter ablation for PAF. Twenty-four hour Holter monitoring was obtained 3–6 months after the catheter ablation. Recurrence was defined as any symptomatic or documented atrial arrhythmia of > 30 s after a 3 month blanking period.

### Statistical analysis

Normality was assessed using the Shapiro–Wilk test. All data are shown as mean ± standard division, median (interquartile range), or number of participants (percentages), as appreciate.

Comparison of the continuous variables between the two groups was performed by independent t tests and Mann–Whitney *U* tests. Categorical variables were assessed using the Chi-square test or Fisher’s exact test, as appropriate. Correlations between each group between continuous indices were assessed using Pearson’s and Spearman’s correlation coefficients for parametric and nonparametric data, respectively. A blinded list of mixed PAF patients and control subjects in random order was used for image analysis. Two independent observers measured LV-ECV and LA strain in 20 randomly selected subjects (10 PAF and 10 control subjects) to assess interobserver reproducibility. Furthermore, one observer measured LV-ECV and LA strain twice with a washout period of 3 months to determine intraobserver reproducibility. The inter- and intraobserver reproducibility of the LV-ECV measurement and LA strain were tested by calculating interclass correlation coefficient (ICC). Values of P < 0.05 were considered significant. Statistical analyses were performed using SPSS version 23 software (SPSS, Chicago, IL).

### Ethics approval and consent to participate

This study was conducted in accordance with the principles of the Declaration of Helsinki and with the approval of the Nihon University Institutional Review Board (approval number RK-180410-02, April 24th, 2018). All participants gave their written informed consent prior to participation.

### Consent for publication

All data used or contained in this study were consented for publication as stipulated by Institutional Review Board policy.

## Results

### Baseline patient characteristics

Of a total of 129 patients with PAF, 23 patients who had severe artifacts due to body or respiratory motions (n = 4), LGE (n = 5), or AF rhythm at the time of study (n = 14) were excluded. The remaining 106 PAF patients were analyzed for this study. Echocardiography was performed in all patients within a week from CMR examination. Demographic data for 106 PAF patients and 20 control subjects are summarized in Table 1. No significant differences in age, sex, heart rate, hematocrit (at the day of CMR examination), medications, lifestyle disease, CHADS_2_ score, or CHA_2_DS_2_-VASc score except BMI were apparent between the groups. Usages of both anticoagulant and antiarrhythmic drugs were more frequent in patients with PAF (*P* < 0.0001, respectively).

**Table 1:** Demographic data of the PAF and control groups.

	PAF (n = 106)	Control (n = 20)	*P* value
Age (years)	66.1 ± 10.7	68.3 ± 8.6	0.50
Male (n, %)	70 (66%)	11 (55%)	0.35
BMI (kg/m^2^)*	24.3 ± 3.7	22.3 ± 2.9	0.04
SBP (mmHg)	132.7 ± 17.7	132.0 ± 18.9	0.87
DBP (mmHg)	77.2 ± 13.8	76.6 ± 10.2	0.86
HR (beats/min)	72.0 ± 14.7	71.6 ± 9.4	0.78
Hematocrit (%)	40.6 ± 4.4	41.5 ± 3.6	0.38
eGFR (mL/min/1.73 m^2^)	73.0 ± 14.2	74.1 ± 22.8	0.27
NT-proBNP (pg/mL)	209.4 ± 333.5	289.2 ± 731.0	0.34
**Medical and drug history**	9 (3–12)	–	
Hypertension (n, %)	64 (60%)	13 (65%)	0.70
Diabetes (n, %)	17 (16%)	5 (25%)	0.33
Dyslipidemia (n, %)	38 (36%)	10 (50%)	0.23
CAD (n, %)	1 (1%)	1 (5%)	0.18
Heart failure (n, %)	7 (7%)	1 (5%)	0.79
Stroke (n, %)	12 (11%)	1 (5%)	0.39
CHADS_2_ score	1.3 ± 1.1	1.2 ± 0.8	0.76
CHA_2_DS_2_-VASc score	2.2 ± 1.4	2.4 ± 1.4	0.50
Duration of PAF (month)	9 (3–12)	–	–
ACEi/ARB (n, %)	37 (35%)	7 (35%)	0.99
Calcium channel blocker (n, %)	39 (37%)	8 (40%)	0.79
Beta-blocker (n, %)	50 (47%)	5 (25%)	0.07
Diuretic (n, %)	8 (8%)	1 (5%)	0.67
Statin (n, %)	33 (31%)	6 (30%)	0.92
Antidiabetic drugs/insulin (n, %)	12 (11%)	0 (0%)	0.11
Anticoagulant drugs (n, %)*	106 (100%)	2 (10%)	< 0.0001
Number of antiarrhythmic drugs*	1.3 ± 0.9	0.2 ± 0.4	< 0.0001

All data are shown as mean ± standard division, median (interquartile range), or number of participants (percentages), as appreciate.

*ACEi* angiotensin-converting enzyme inhibitor, *ARB* angiotensin II receptor blocker, *BMI* body mass index, *CAD* coronary artery disease, *DBP* diastolic blood pressure, *eGFR* estimated glomerular filtration rate, *HR* heart rate, *NT-proBNP* N terminal pro B type natriuretic peptide, *PAF* paroxysmal atrial fibrillation, *SBP* systolic blood pressure. *P < 0.05.

### LV function and strain

The results of LV function and strain between the groups are shown in Table 2. No significant differences were seen in LV volumetric or functional parameters. Global radial strain, global circumferential strain, and global longitudinal strain did not differ significantly between the groups.

**Table 2:** Comparison of left ventricular function and strain between the PAF and control groups.

	PAF (n = 106)	Control (n = 20)	*P* value
**LV function**			
LVEF (%)	61.1 ± 4.8	62.2 ± 4.0	0.35
LVEDVI (mL/m^2^)	74.1 ± 14.5	74.8 ± 12.5	0.84
LVESVI (mL/m^2^)	29.0 ± 7.4	28.6 ± 7.3	0.60
LVSVI (mL/m^2^)	45.2 ± 8.9	46.3 ± 6.3	0.61
LVMI (g/m^2^)	44.2 ± 8.4	45.9 ± 6.9	0.30
**LV strain (%)**			
GRS	29.6 ± 8.1	32.9 ± 9.6	0.27
GCS	− 17.3 ± 3.2	− 18.3 ± 3.3	0.22
GLS	− 12.7 ± 2.3	− 13.2 ± 2.5	0.35

All data are shown as mean ± standard deviation.

*GCS* global circumferential strain, *GLS* global longitudinal strain, *GRS* global radial strain, *LVEDVI* left ventricular end diastolic volume index, *LVEF* left ventricular ejection fraction, *LVESVI* left ventricular end systolic volume index, *LVMI* left ventricular mass index, *LVSVI* left ventricular stroke volume index.

### LA phasic function and strain

**Figure 2:**
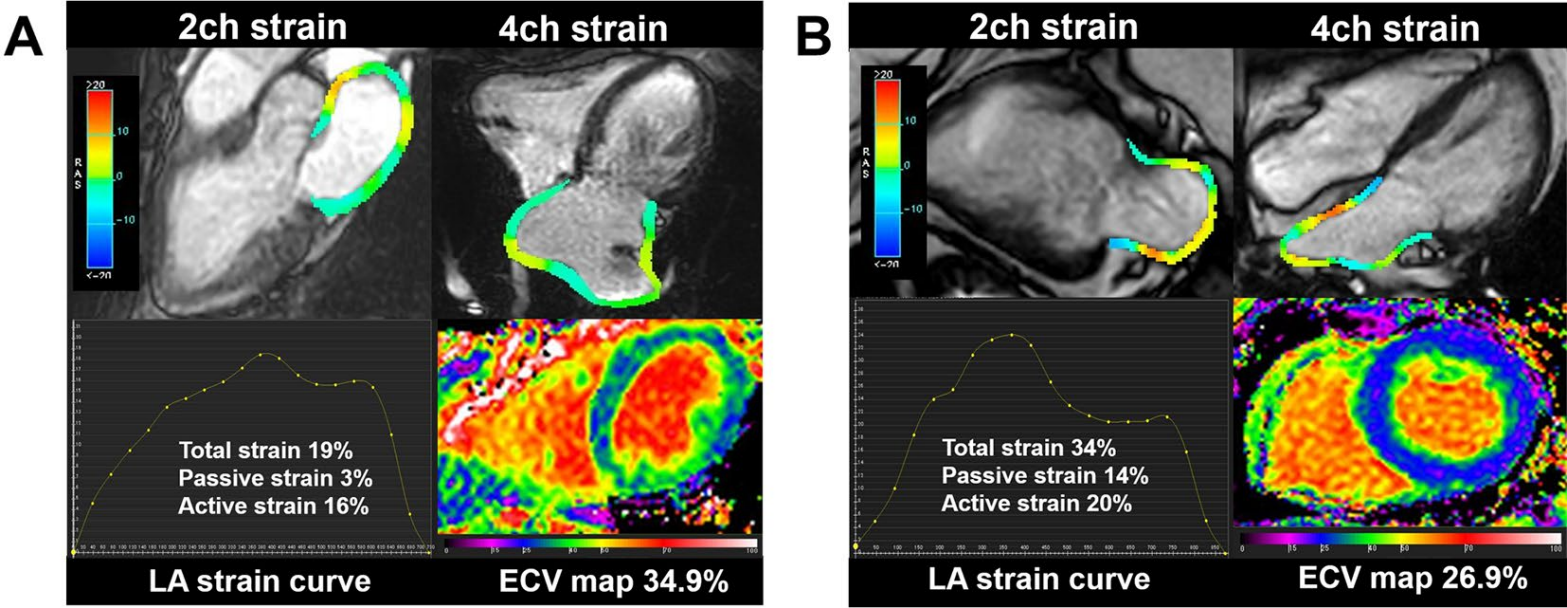
Representative imaging findings in a patient with PAF (**A**) and a control subject (**B**). LV-ECV is higher in the patient with PAF (34.9%) than in the control subject (26.9%), indicating advanced myocardial fibrosis. LA strain analysis reveals that total and passive strain is substantially lower in PAF (total strain: 19% vs. 34%, passive strain: 3% vs. 14%), while active strain is similar between the patient with PAF and the control subject (16% vs. 20%).

The results of LA phasic function and strain are summarized in Table 3. Representative cases of a patient with PAF and a control subject are shown in Fig. 2. LAVI (V_max_, V_pre-ac_, V_min_) was significantly greater in the PAF group than in the control group (LAVI_max_: 43.6 ± 17.6 vs. 35.6 ± 18.0 mL/m^2^, *P* = 0.01; LAVI_pre-ac_: 34.6 ± 15.4 vs. 26.1 ± 16.4 mL/m^2^, *P* = 0.002; LAVI_min_: 23.9 ± 14.5 vs. 17.6 ± 16.9 mL/m^2^, *P* = 0.004). Total and passive LAEF and LA strain (corresponding to reservoir and conduit functions) were significantly decreased in the PAF group than in the control group (total LAEF: 48.0 ± 10.7 vs. 55.4 ± 13.1%, *P* = 0.004; passive LAEF: 21.5 ± 7.3 vs. 29.0 ± 7.5%, *P* < 0.0001; total LA strain: 22.3 ± 7.6 vs. 29.3 ± 10.2%, *P* < 0.0001; passive LA strain: 10.9 ± 4.6 vs. 14.1 ± 5.8%, *P* = 0.002). Active LAEF (corresponding to booster pump function) did not differ between the PAF and control groups (33.9 ± 10.9% vs. 37.9 ± 13.3%, *P* = 0.15), while active LA strain was significantly decreased in the PAF group (11.4 ± 4.3 vs. 15.2 ± 5.6%, *P* = 0.002).

**Table 3:** Comparison of left atrial function and strain between the PAF and control groups.

	PAF (n = 106)	Control (n = 20)	*P* value
**LAVI (mL/m**^**2**^**)**			
LAVI_max_	43.6 ± 17.6	35.6 ± 18.0	0.01
LAVI_pre-ac_	34.6 ± 15.4	26.1 ± 16.4	0.002
LAVI_min_	23.9 ± 14.5	17.6 ± 16.9	0.004
**Reservoir function**			
Total LAEF (%)	48.0 ± 10.7	55.4 ± 13.1	0.004
Total LA strain (%)	22.3 ± 7.6	29.3 ± 10.2	< 0.0001
SRt (s^−1^)	1.1 ± 0.4	1.6 ± 0.7	< 0.0001
**Conduit function**			
Passive LAEF (%)	21.5 ± 7.3	29.0 ± 7.5	< 0.0001
Passive LA strain (%)	10.9 ± 4.6	14.1 ± 5.8	0.002
SRp (s^−1^)	− 0.9 ± 0.4	− 1.1 ± 0.5	0.02
**Booster pump function**			
Active LAEF (%)	33.9 ± 10.9	37.9 ± 13.3	0.15
Active LA strain (%)	11.4 ± 4.3	15.2 ± 5.6	0.002
SRa (s^−1^)	− 1.2 ± 0.5	− 1.7 ± 0.7	< 0.0001

All data are shown as mean ± standard deviation.

*LAEF* left atrial empty fraction, *LAVI* left atrial volume index, *max* maximum, *min* minimum, *pre-ac* pre atrial contraction, *SR* strain rate.

### LV-ECV

**Figure 3:**
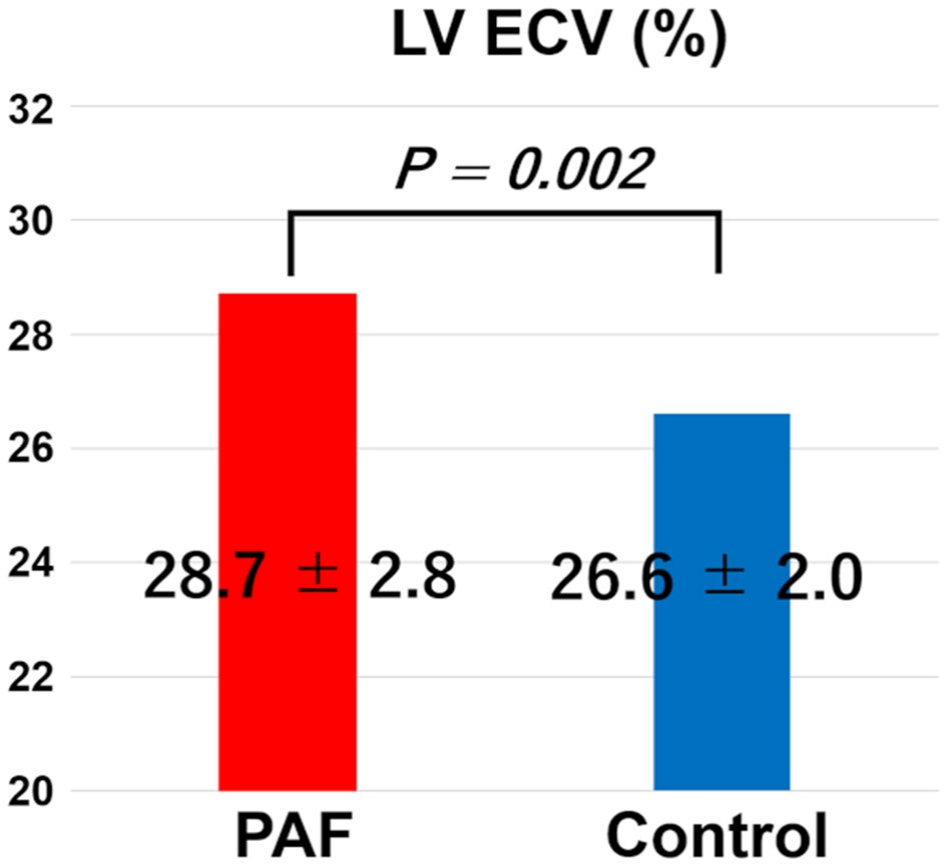
Differences in LV-ECV between the PAF and control groups. LV-ECV is significantly higher in the PAF group (28.7 ± 2.8%) than in the control group (26.6 ± 2.0%, *P* = 0.002).

LV-ECV was significantly greater in the PAF group (28.7 ± 2.8%) than in the control group (26.6 ± 2.0%, *P* = 0.002) (Fig. 3).

### Correlation between LV-ECV and various parameters in PAF patients

Correlations between LV-ECV and various parameters in PAF patients are summarized in Table 4. In the PAF group, LV-ECV correlated significantly with NT-proBNP (*r* = 0.33, *P* = 0.001), E/e′ (*r* = 0.30, *P* = 0.002), LAVI (LAV_max_: *r* = 0.31, *P* = 0.001, LAV_pre-ac_: *r* = 0.32, *P* = 0.001; LAV_min_: *r* = 0.30, *P* = 0.002), and number of antiarrhythmic drugs (*r* = 0.20, *P* = 0.04), but not with LV mass index (*r* = − 0.08, *P* = 0.42), LVEF (*r* = − 0.02, *P* = 0.85), or PAF duration (*r* = − 0.09, *P* = 0.40). Regarding LA strain, correlations were apparent between LV-ECV and LA reservoir function (total LA strain: *r* = − 0.21, *P* = 0.03) and LA conduit function (passive LA strain: *r* = − 0.25, *P* = 0.009), while no correlation with LA booster pump function (active LA strain: *r* = − 0.09, *P* = 0.38) was evident in PAF patients.

**Table 4:** Correlation of LV-ECV in PAF patients.

Parameters	LV-ECV	
	*r*	*P* value
Age	0.18	0.06
NT-proBNP*	0.33	0.001
LVEF	− 0.019	0.85
LVMI	− 0.08	0.42
E/e′*	0.30	0.002
LAVI_max_*	0.31	0.001
LAVI_pre-ac_*	0.32	0.001
LAVI_min_*	0.30	0.002
PAF duration	− 0.09	0.40
Number of antiarrhythmic drugs*	0.20	0.04
**Reservoir function**		
Total LAEF (%)*	− 0.19	0.05
Total LA strain (%)*	− 0.21	0.03
SRt (s^−1^)	− 0.17	0.08
**Conduit function**		
Passive LAEF (%)	− 0.18	0.07
Passive LA strain (%)*	− 0.25	0.009
SRp (s^−1^)*	0.28	0.004
**Booster pump function**		
Active LAEF (%)	− 0.14	0.15
Active LA strain (%)	− 0.09	0.38
SRa (s^−1^)	0.14	0.16

*ECV* extracellular volume fraction, *LAEF* left atrial empty fraction, *LAVI* left atrial volume index, *LV* left ventricular, *LVEF* left ventricular ejection fraction, *LVMI* left ventricular mass index, *NT-proBNP* N terminal pro B type natriuretic peptide, *PAF* paroxysmal atrial fibrillation, *SR* strain rate. *P < 0.05.

### Correlation between the LA strain and heart rate and CHA_2_DS_2_-VASc score in PAF patients

**Figure 4:**
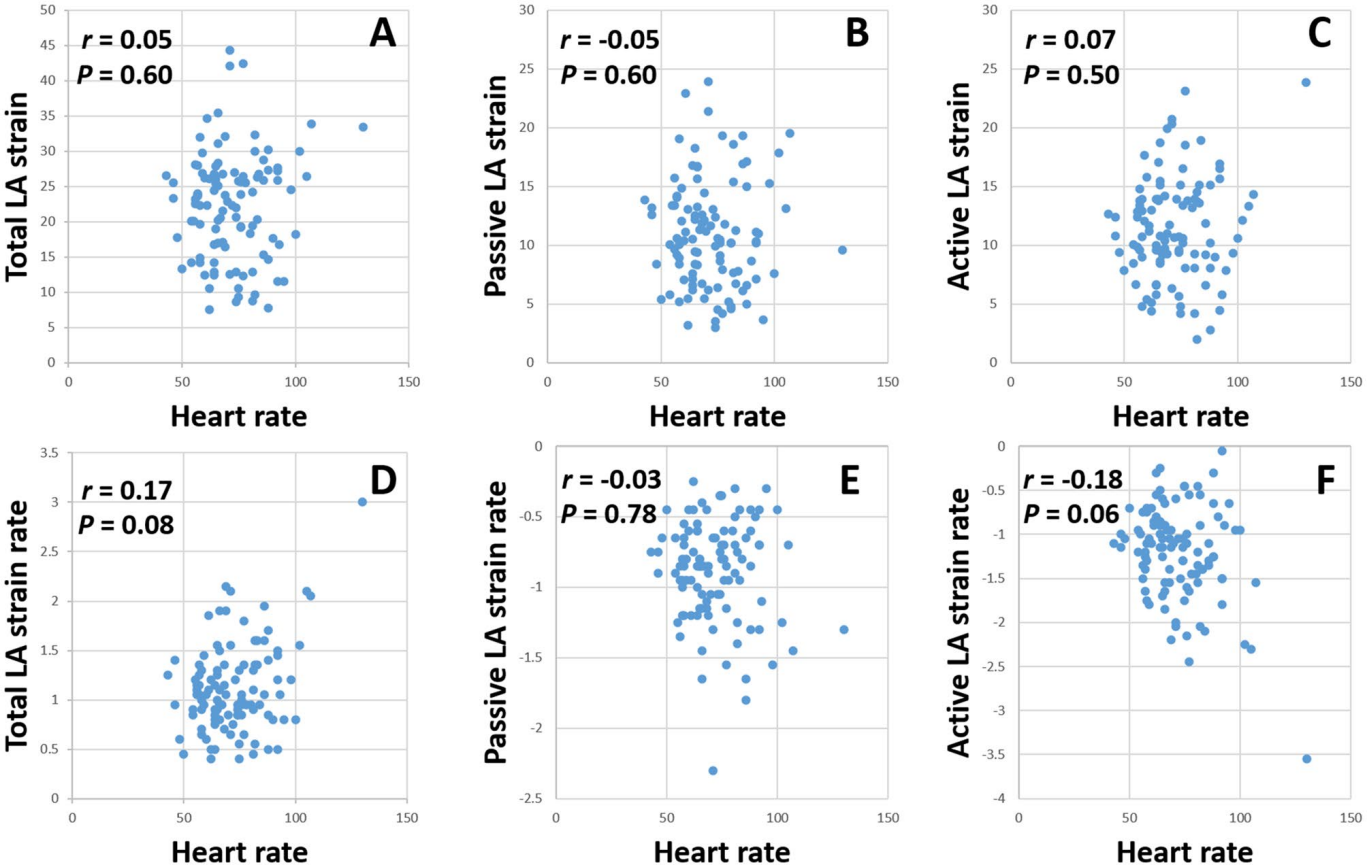
Correlation between the heart rate and (**A**) total LA strain, (**B**) passive LA strain, (**C**) active LA strain, (**D**) total LA strain rate, (**E**) passive LA strain rate, and (**F**) active LA strain rate.

The LA strain and strain rate in PAF patients did not correlate significantly with the heart rate (total LA strain: *r* = 0.05, *P* = 0.60, total LA strain rate: *r* = 0.17, *P* = 0.08, passive LA strain: *r* = − 0.05, *P* = 0.60, passive LA strain rate: *r* = − 0.03, *P* = 0.78, active LA strain: *r* = 0.07, *P* = 0.50, active LA strain rate: *r* = − 0.18, *P* = 0.06). The scatter plots between the LA strain, strain rate, and heart rate are shown in Fig. 4. The passive LA strain correlated negatively with the CHA_2_DS_2_-VASc score (*r* = *−* 0.27,* P* = 0.005) but the total (*r* = *−* 0.18, *P* = 0.07) and active LA strain (*r* = *−* 0.08,* P* = 0.41) did not.

### Postablation outcomes

During the median follow-up period of 7 (4, 10) months, 5 patients (5%) experienced AF recurrences. There were no significant differences in the CMR cardiac parameters including the LV-ECV (28.5 ± 2.3 vs. 28.7 ± 2.8%, *P* = 0.83), LAVI_max_ (53.4 ± 18.9 vs. 43.2 ± 17.4 mL, *P* = 0.34), LAEF_total_ (47.7 ± 10.5 vs. 48.0 ± 10.7%, *P* = 0.96) and LA strain_total_ (22.3 ± 7.5 vs. 22.3 ± 7.6%, *P* = 0.99) between the recurrent AF group and no recurrent AF group.

### Intra- and interobserver reproducibility

ICC for the intra- and interobserver measurements of LV-ECV showed almost-perfect agreement. All LA strain and SR parameters displayed good to excellent reproducibility for both intra- and interobserver classes (Table 5).

**Table 5:** Intraobserver and interobserver reproducibility for LV-ECV and LA strain parameters.

	ICC (95% CI)	
	Intraobserver	Interobserver
LV-ECV	0.98 (0.96–0.99)	0.98 (0.95–0.98)
LA total strain	0.88 (0.69–0.95)	0.86 (0.65–0.95)
LA passive strain	0.89 (0.71–0.96)	0.78 (0.45–0.91)
LA active strain	0.88 (0.69–0.95)	0.80 (0.49–0.92)
LA SRt	0.76 (0.40–0.91)	0.73 (0.32–0.89)
LA SRp	0.89 (0.73–0.96)	0.81 (0.52–0.93)
LA SRa	0.86 (0.64–0.94)	0.79 (0.47–0.92)

*ECV* extracellular volume fraction, *ICC* intraclass correlation coefficient, *LA* left atrial, *LV* left ventricular, *SRa* peak late negative strain rate, *SRp* peak early negative strain rate, *SRt* peak positive strain rate.

## Discussion

This study disclosed the difference in the extent of LV myocardial fibrosis and LA functional remodeling by the simultaneous assessment of LV-ECV and feature tracking LA strain derived from a single CMR study between PAF patients and control subjects. The main findings of this study were: (1) CMR-FT LA strain could identify subtle LA booster pump dysfunction undetectable by conventional active LAEF; (2) LV-ECV was significantly greater in PAF patients than in control subjects, indicating advanced LV myocardial fibrosis; (3) significant correlations were evident between LV-ECV and LA reservoir function and LA conduit function in the PAF group; (4) LV-ECV and LA strain parameters displayed robust reproducibility for both intra- and interobserver classes.

### LA remodeling

LA remodeling involves structural and functional changes, and those changes in LA already coexist before the development and persistence of AF^23^. Early detection of these changes in individuals is helpful for optimal management of AF in clinical practice. LA dilation reflected by LA structural remodeling is a well-known risk for AF development and stroke^24,25^. As expected, our study showed LA volumes were significantly higher in the PAF group than in the control group. Nonetheless, in our cohort, the percentage of LA dilation in PAF was relatively low (26%) and mean LAV_max_ (43.6 ± 17.6 mL/m^2^) was within the normal range (26–52 mL/m^2^)^26^. Given the complex atrial geometry and thin atrial wall thickness, cardiovascular imaging for noninvasive assessment of LA function is challenging. With the remarkable developments in CMR imaging (excellent spatial resolution to identify heterogeneous atrial wall thickness), complex LA function can now be accurately assessed^12^. LA function has been divided into three components:^21^ reservoir function when the atria store blood in systole, as a conduit when blood flows passively into the LV in early diastole, and as a booster pump when the atria contract in late diastole. In our study, total and passive LAEF (corresponding to reservoir and conduit functions) were decreased in the PAF group as compared to the control group, in line with a previous report^23^. However, our study demonstrated that active LAEF (corresponding to booster pump function) did not differ significantly between the PAF and control groups. Whether active LAEF is reduced^23^ or not^27^ in PAF patients has remained controversial. In an animal experiment, AF itself induced booster pump dysfunction by causing a tachycardia-induced atrial cardiomyopathy^28^. Shin et al. demonstrated that active LAEF was significantly reduced in patients with frequent episodes of AF than in others^29^. Alternatively, even in patients with PAF, booster pump function tends to be unaffected when the basic cardiac rhythm is almost sinus rhythm. A potential explanation for similar active LAEF between the PAF and control groups in our study was that the frequency of PAF episodes had been low and the duration of PAF had been short, since we selected PAF patients presenting with sinus rhythm at both outpatient clinic and CMR examinations. LA remodeling in the PAF group might thus be mild and booster pump function assessed by LAEF was preserved in the present study.

Myocardial strain is more sensitive imaging marker to detect early changes of cardiac function than EF, as is the case with incipient disease such as PAF^30^. Habibi et al. conducted an observational study to examine the association between the LA function using CMR-FT LA strain (total LA strain and 3 phasic LA strain rate,) and 3-dimensional LA LGE (LA fibrosis) in heterogeneous AF patients (both PAF and persistent AF)^31^. In that study, the LA functional parameters and LA LGE were compared between paroxysmal and persistent AF or mixed AF (both paroxysmal and persistent) patients and healthy volunteers. They concluded that an increased LA LGE was associated with a decreased LA phasic function, and the assessment of the LA function by the CMR-FT LA strain may add important information about the physiological importance of LA fibrosis. On the other hand, our study evaluated all 3 phasic LA strain and LA strain rates (total, active, and passive) in PAF patients and directly compared PAF patients with control subjects to explore the early changes in the LA remodeling, which was undetectable by the conventional LA phasic function. Our study revealed that CMR-FT LA strain could identify subtle LA booster pump dysfunction undetectable by conventional active LAEF. Our study found no correlation between the LA strain/strain rate and heart rate, as a conflicting result to a previous study reported by Goldberg et al. in which a curvilinear increase in the LA strain with a longer RR interval (*r* = 0.45, *P* < 0.0001) was found^32^. In our cohort, 90 out of 106 PAF patients (84%) took antiarrhythmic drugs with negative inotropic effects and more than half of them (56%) were prescribed beta-blockers. Sardana et al. reported that beta-blocker use was significantly associated with an impaired LA strain^33^. The potential explanation for no correlation between the LA strain and heart rate is that the high proportion of beta-blocker use with a wide range of doses might have cancelled the increase in the strain with a lowering of the heart rate. Consequently, the high proportion of negative inotropic medicine (mainly betablockers) use with various types and doses may have resulted in a loss of the entire correlation between the LA strain and heart rate in this study. Additionally, the correlation between the LA strain and heart rate was established in normal conditions in the previous studies^32,34^. The difference in the LA between normal and PAF patients (LA functional and anatomical remodeling) may lead to conflicting results. Another informative finding of our study was that the CHA_2_DS_2_-VASc score correlated negatively with the passive LA strain (*r* = *− *0.27,* P* = 0.005) but did not correlate with the total (*r* = *− *0.18, *P* = 0.07) and active LA strain (*r* = *− *0.08,* P* = 0.41). Those findings added to the previous results by Ahmed et al. that the total LA strain did not correlate with the CHA_2_DS_2_-VASc score^35^. Patients with a high CHA_2_DS_2_-VASc score are likely to have advanced systemic atherosclerosis and advanced systemic atherosclerosis tends to cause a higher LV end-diastolic pressure. The higher LV end-diastolic pressure may preferentially affect the conduit function.

A recent study showed LA strain to be the strongest independent predictor of progression to persistent AF in a model including LA diameter, volume, and function^36^. In this context, a large registry demonstrated that LA strain and p-wave-to-A’ duration on echocardiographic tissue Doppler imaging was independently associated with stroke risk in a model including CHA_2_DS_2_-VASc score, age, and anticoagulant use^37^. Moreover, emerging data suggest an independent, inverse association between LA strain measured using CMR and incident heart failure^38^.

### LV remodeling

A histological study by Frustaci et al. showed that LV endomyocardial biopsy in 14 lone AF patients demonstrated nonspecific necrosis or fibrosis in 60%^39^. We have previously demonstrated that ventricular fibrotic changes are more pronounced in AF patients than in subjects with sinus rhythm according to echocardiography-derived integrated backscatter^40^. CMR-based myocardial ECV has been regarded as the most robust noninvasive measurement for quantifying myocardial fibrosis^7,8^. However, evidence for the association between LV-ECV and AF remains limited. Neilan et al. previously reported that patients with hypertension and AF had an increased LV-ECV as compared to healthy control patients, and an expansion of the LV-ECV was a strong predictor of recurrent AF^41^. The difference between our study and Neilan’s study is the patient selection. Neilan’s study focused on hypertensive patients with AF, while we recruited consecutive PAF patients with or without hypertension. Additionally, the previous CMR studies regarding AF evaluated LA remodeling and LV remodeling separately, while our study evaluated LA remodeling and LV remodeling concurrently in a single clinical CMR examination.

In our study, CMR-based LV-ECV revealed that myocardial fibrosis in the setting of PAF was more advanced than in control subjects. The pathogenesis of LV fibrosis in PAF has not been fully elucidated. In our study, the LV-ECV did not correlate with the PAF duration (*r* = − 0.09, *P* = 0.40). LV fibrosis may occur secondary to AF as a consequence of rapid ventricular rates or the irregularity of ventricular contraction^42,43^. It seems that the main contributor to the LV-ECV was not the PAF duration but the frequency of PAF episodes. Additionally, the LV-ECV correlated positively with the number of antiarrhythmic drugs (*r* = 0.20*, P* = 0.04). The patients taking multiple antiarrhythmic drugs were prone to frequent episodes of AF. As a consequence, the rapid ventricular rates or irregularity of the ventricular contractions may have resulted in an elevation of the LV-ECV. We also found a significant correlation between LV-ECV and LA volume index in PAF patients. Elevated LV-ECV is reported to be a major contributor to the impaired LV relaxation and stiffness^44^. With increased LV stiffness, left atrial pressure rises to maintain adequate ventricular filling, and the increased atrial wall tension leads to subsequent left atrial enlargement. Furthermore, LV-ECV in PAF patients significantly correlated with LA reservoir function and LA conduit function, suggesting a potential link between LV remodeling and LA functional remodeling.

### Limitations

This study has some limitations that need to be acknowledged when interpreting the results. First, LV-ECV and LA/LV strain data in our study were derived from a single center and a single vendor. All patients were referred for PVI ablation. This group may not be representative of all patients with PAF. A multicenter, multivendor, and heterogeneity study is warranted to validate the results of our study. Second, subjects in this study were ineligible for cardiac catheterization including endomyocardial biopsy and pressure study, and histological validation and physiological parameters regarding atrial and ventricular pressure were not obtained. However, a number of studies have shown myocardial ECV as determined by CMR correlated excellently with histological quantification of myocardial fibrosis and association between atrial pressure and atrial remodeling^45^. Third, the association of LA strain and electrophysiological parameters was not analyzed. Moreover, the impact of PVI for PAF on LA/LV remodeling was also undetermined, and continued research is required regarding these points. Fourth, it is challenging to assess whether the LA remodeling precedes the LV remodeling or vice versa due to a lack of series imaging data of the LA and LV changes. Fifth, only a 2-dimensional LA strain analysis was performed due to no 3-dimensional technique with CMR feature tracking being available, and the LA motion is complex and there may be missing regional motion in 2-dimensional views during the cardiac cycle. Finally, the follow-up period was relatively too short to identify the recurrence of AF and wearable continuous electrocardiogram devices did not apply for the detection of AF recurrences in this study. These issues might have underestimated the actual recurrences and results of no significant differences in the CMR cardiac parameters including the LV-ECV, LA volume, LAEF, and LA strain between the recurrent AF group and no recurrent AF group in this study. A further study with a long follow-up period and wearable continuous electrocardiogram devices is warranted.

## Conclusion

CMR-FT LA strain in combination with the LV-ECV in a single clinical study offers a potential imaging marker that identifies LA/LV remodeling including subtle LA booster pump dysfunction undetectable by the conventional booster pump LAEF in the PAF stage and could be a valuable tool for clinicians.

## Supplementary Information

Supplementary Information.

## Data Availability

The datasets used and/or analyzed during the current study available from the corresponding author on reasonable request.

## Abbreviations

AF: Atrial fibrillation
BMI: Body mass index
CMR: Cardiovascular magnetic resonance
ECV: Extracellular volume fraction
EF: Emptying fraction
FT: Feature tracking
ICC: Interclass correlation coefficient
LA: Left atrial/atrium
LAV: Left atrial volume
LAVI: Left atrial volume index
LGE: Late gadolinium enhancement
LV: Left ventricular
MOLLI: Modified look-Locker inversion recovery sequence
PAF: Paroxysmal atrial fibrillation
pre-ac: Pre-atrial contraction
PVI: Pulmonary vein isolation
SR: Strain rate
SSFP: Standard steady-state free precession
