# Supplementary material for: Comprehensive assessment of left atrial and ventricular remodeling in paroxysmal atrial fibrillation by the cardiovascular magnetic resonance myocardial extracellular volume fraction and feature tracking strain
Source: Sci Rep. 2021 May 25;11:10941. doi: 10.1038/s41598-021-90117-6 (PMC8149643; doi:10.1038/s41598-021-90117-6)
Supplement: Supplementary file 1 — Supplementary Information. [file 41598_2021_90117_MOESM1_ESM.docx]

**Comprehensive assessment of left atrial and ventricular remodeling in paroxysmal atrial fibrillation by cardiovascular magnetic resonance myocardial extracellular volume fraction and feature tracking strain**

Akimasa Yamada, Naoki Hashimoto, Hidesato Fujito, Takumi Hatta, Yuki Saito, Naoto Otsuka, Yuji Wakamatsu, Masaru Arai, Ryuta Watanabe, Sayaka Kurokawa, Daisuke Kitano, Koichi Nagashima, Shunichi Yoda,Yasuo Okumura*

Department of Cardiovascular Medicine, Nihon University Graduate School, Tokyo, Japan

*Corresponding author: Yasuo Okumura, MD

Department of Cardiovascular Medicine, Nihon University Graduate School

30-1 Oyaguchi kamimachi, Itabashi, Tokyo 173-8610, Japan

E-mail: okumura.yasuo@nihon-u.ac.jp

Tel: +81-3-3972-8111

Fax: +81-3-3972-1098

**Supplementary materials**

Detailed CMR sequence parameters.

The standard SSFP cine images were acquired with the typical parameters: slice thickness: 8 mm, slice gap: 1 mm, TR/TE: 3.4/1.7 ms, flip angle 60°, field of view: 250×250 mm^2^, acquisition matrix size: 156×156, reconstruction matrix size: 320×320 SENSE factor 2, temporal resolution < 40 ms. LGE images were obtained a T1-weighted inversion-recovery gradient echo technique (slice thickness 10 mm, slice gap: -5 mm, TR/TE = 4.6/2.2 ms, flip angle 15°, field of view 360×360 mm^2^, acquisition matrix size 266×180, reconstruction matrix size: 384×384, SENSE factor 2, inversion time 200 to 300 ms). Pre and post T1 measurements were acquired with the following parameters: slice thickness 10 mm, slice gap: 10 mm, TR/TE = 2.7/1.8 ms, flip angle 35°, field of view 300×300 mm^2^, acquisition matrix size 142×142, reconstruction matrix size: 256×256, SENSE factor 2, 11 images from three inversions (3+3+5) with three heartbeats pauses prior to the second and third inversions and an adiabatic prepulse.
